# Cancer diagnosed by emergency admission in England: an observational study using the general practice research database

**DOI:** 10.1186/1472-6963-13-308

**Published:** 2013-08-14

**Authors:** Carmen Tsang, Alex Bottle, Azeem Majeed, Paul Aylin

**Affiliations:** 1 Dr Foster Unit at Imperial, Ground Floor 3 Dorset Rise, EC4Y 8EN, London, England; 2 Department of Primary Care and Public Health, Imperial College London, 3rd Floor Reynolds Building, St Dunstan’s Road, London W6 8RP, England

**Keywords:** Cancer, Primary care, Emergency admissions, Diagnosis, Risk factors, Patient records

## Abstract

**Background:**

Patients diagnosed with cancer by the emergency route often have more advanced diseases and poorer outcomes. Rates of cancer diagnosed through unplanned admissions vary within and between countries, suggesting potential inconsistencies in the quality of care. To reduce diagnoses by this route and improve patient outcomes, high risk patient groups must be identified. This cross-sectional observational study determined the incidence of first-ever diagnoses of cancer by emergency (unplanned) admission and identified patient-level risk factors for these diagnoses in England.

**Methods:**

Data for 74,763 randomly selected patients at 457 general practices between 1999 and 2008 were obtained from the General Practice Research Database (GPRD), including integrated Hospital Episode Statistics (HES) data and Office for National Statistics (ONS) mortality data. The proportion of first-ever diagnoses by emergency admission out of all recorded first cancer diagnoses by any route was analysed by patient characteristics.

**Results:**

Diagnosis by emergency admission was recorded in 13.9% of patients diagnosed with cancer for the first time (n = 817/5870). The incidence of first cases by the emergency route was 2.51 patients per 10,000 person years. In adjusted regression analyses, patients of older age (p < 0.0001), living in the most deprived areas (RR 1.93, 95% CI 1.51 to 2.47; p < 0.0001) or who had a total Charlson score of 1 compared to 0 (RR 1.34, 95% CI 1.06 to 1.69; p = 0.014) were most at risk of diagnosis by emergency admission. Patients with more prior (all-cause) emergency admissions were less at risk of subsequent diagnosis by the emergency route (RR 0.31 per prior emergency admission, 95% CI 0.20 to 0.46; p < 0.0001).

**Conclusions:**

A much lower incidence of first-ever cancer diagnoses by emergency admission was found compared with previous studies. Identified high risk groups may benefit from interventions to reduce delayed diagnosis. Further studies should include screening and cancer staging data to improve understanding of delayed or untimely diagnosis and patient care pathways.

## Background

Improving cancer outcomes is an international health priority, including the reduction of diagnoses through the emergency route. Patients who are diagnosed by emergency (or unplanned) admission typically have more advanced diseases and poorer outcomes such as survival, especially older patients [1-5]. The term “emergency” is often used synonymously with “unplanned” admissions and is applied as such in this paper. Emergency admissions are characterised as arising through Accident and Emergency (A&E) services, general practitioner (GP), bed bureau, outpatient clinic or other means which may include the A&E department of another care provider [6]. To reduce delayed or untimely diagnoses, factors influencing patients’ access to services before diagnosis, including pre-referral general practice consultations, must be better understood and especially in high risk patient groups [7,8].

Approximately 24% of cancers are diagnosed through the emergency route in England [9-11]. However, the rate of these admissions varies across the country, suggesting that there are inconsistencies and delays in local services [4,9,12]. Using national data from hospitals in England (Hospital Episode Statistics, HES), we previously identified patient and general practice characteristics associated with increased risk of cancer diagnosis by emergency admission [11]. When data from only one care setting are analysed, inclusion of false positive cases or incomplete capture of cases may occur [11,13,14]. Elliss-Brookes et al. (2012) used HES linked with cancer registry, cancer screening and cancer waiting times data to examine routes to diagnosis, but their study period was only two years between 2006 and 2008 [10]. In our current study, the accuracy of case ascertainment (correct identification of patients with a first-ever emergency admission for cancer) was improved by linking hospital data with primary care data for a ten-year study period.

Studies in England have described patient risk factors for diagnosis by emergency admission for some cancers, such as breast, colorectal, oesophageal, gastric and lung cancers [4,13]. Unlike previous research focusing on specific types, this study examined all cancers. We investigated predictors of first-ever emergency admissions for cancer, as a proxy for first-ever delayed or untimely diagnosis by the emergency route. We also determined the incidence of these admissions from first-ever cancer diagnoses by any diagnosis route.

## Methods

### General practice research database

National data were obtained from the General Practice Research Database, GPRD (superseded by the Clinical Practice Research Datalink, CPRD, since April 2012), under a Medical Research Council (MRC) licence for academic institutions. The GPRD is one of the largest electronic patient-level, anonymised primary care databases in the world. It includes clinical and non-clinical data, which can be linked with other data sources such as disease registries. It is renowned for its representational coverage of the English population and is well validated for epidemiological and biomedical research, including studies on cancer [15-20]. GPRD performs preliminary data cleaning to ensure that the data are of a consistent acceptable standard for research purposes (up-to-standard, UTS). The study population was drawn from patients whose records were deemed “acceptable” and who were registered at practices with an UTS date during the study period. Data were extracted from the October 2010 build of the GPRD database.

### Integrated dataset

Read codes are the current standard clinical classification system for English primary care, consisting of a comprehensive set of medical and non-medical terms within a structured hierarchy [21]. The Unified 5-Byte Version 2 Read codes recorded in GPRD can be mapped to ICD-10 codes, which are used in secondary care and recorded in HES [21]. Cross-mapping of Read and ICD-10 codes was performed using the NHS Health and Social Care Information Centre’s NHS Clinical Terminology Browser Version 1.04.

Integrated emergency admissions data from HES, central mortality data from the Office for National Statistics (ONS) and patient-level social deprivation by Index of Multiple Deprivation (IMD) 2007 scores were available in the dataset. Patient-level linkage by National Health Service (NHS) number, date of birth and sex was carried out internally by GPRD before the anonymised dataset was distributed to the authors.

The raw GPRD dataset contained records for 100,000 patients registered at 584 participating general practices during the study period (1st January 1999 to 31st December 2008). Patients with invalid or missing sex, registration date, year of birth or place of residence (not in England) were excluded from analyses. The majority of exclusions were due to non-English residence. Patients recorded as residing in Northern Ireland, Scotland and Wales were excluded as HES data only includes admissions to hospitals in England. The cleaned study dataset contained records for 74,763 patients who were registered at 457 general practices between 1999 and 2008.

### Study sample

Eligible patients were those who had a first-ever cancer diagnosis recorded in general practice records or secondary care (HES) between 1st January 1999 (or first consultation date, whichever occurred last) and 31st December 2008 (or date of transfer out of the practice or death, whichever occurred first). Where patients had one or more transfers out of a general practice, only data from the first registration period were included.

### Cases of first-ever diagnosed cancer

Cancers diagnosed by emergency admission were defined by ICD-10 codes (C codes) and assigned into one of the 22 cancer categories used by the National Cancer Intelligence Network (NCIN), which are also based on ICD-10 codes [22]. Patients diagnosed with cancer by non-emergency routes were identified by a recorded primary care cancer diagnosis (Read Codes, B0-B6) and who did not have a HES record of an emergency admission for cancer whose date preceded the primary care record. Case ascertainment was achieved using the GPRD-generated patient identifier for linkage between the datasets.

Patients diagnosed with other malignant neoplasms (non-melanoma) of the skin or multiple site neoplasms were excluded (Read B33 and ByuE codes, ICD-10 C44 and C97 codes, respectively) (see Additional file 1: Table S1). To improve the accuracy of identifying first-ever diagnoses, we tracked back to the patient’s first record in the study dataset obtained from the GPRD. The frequency of cancer types by diagnosis route (emergency versus non-emergency routes) was explored by a crude match of ICD-10 to Read codes (see Additional file 2: Table S2). Patients with a recorded valid Read or ICD-10 cancer code before the study period were excluded.

### Patient and practice characteristics

Except for the geographical region of the general practice (categorised into Strategic Health Authorities), no other practice characteristics were available in the GPRD dataset. Therefore, mainly patient variables were used: age at diagnosis; sex; ethnicity; deprivation status; comorbidities; continuity of care; follow-up time; length of time registered at the practice; the number of consultations at the general practice, by telephone or home visit with a GP or nurse; referrals and admissions before diagnosis; and practice region. Ethnicity and social deprivation details were only available through the linked HES data for patients who had at least one recorded (any cause) emergency admission.

Comorbidity was measured by a Read code adaptation of the Charlson Index by Khan et al. (2010), derived from Deyo et al’s modified version using ICD-9-CM codes [23]. Each patient’s cumulative comorbidity score can be estimated from predefined weights for certain diseases and conditions included in the Index. A three-banded aggregation of patients’ total Charlson Index score was applied, grouped as score of 0, 1 and at least 2. Continuity of care was measured by the Bice and Boxerman’s Continuity of Care (COC) Index [24]. This Index takes into account the total number of consultations (at the general practice or by telephone) as well as the number of visits to each clinician (GPs or nurses) and the number of clinicians consulted over a fixed time period [24]. Follow-up time was defined as the length of time (in years) between study entry (1st January 1999 or first consultation date, whichever occurred last) and the first recorded diagnosis of cancer (before or on 31st December 2008, or date of transfer out of the practice or death, whichever occurred first).

### Statistical methods

The Mann Whitney U test was used in preliminary descriptive analyses of non-normally distributed ordinal data. Crude and adjusted analyses were performed by modified Poisson regression, with a log link function to generate relative risks. Pre-empting potential problems of misclassification and effect overestimation due to the application of Poisson regression for a binary outcome, the Generalized Estimating Equation (GEE) method was used [25-28]. This approach also accounted for clustering of patients at general practices [26]. The stepwise method was used to select variables for retention in the adjusted model. An advantage of stepwise selection is the reduction of multicollinearity, which in this study may occur due to variables such as age, time at practice and follow up time; as well as prior consultations and referrals. To determine entry and stay in this model, a p-value of 0.05 was used as the cut-off. All analyses were performed in SAS (version 9.2) and regression analyses were performed using the “PROC GENMOD” procedure.

### Ethics approval

The GPRD Group has Trent Multi-Centre Research Ethics Committee approval for all observational research using GPRD data (reference: 05/MRE04/87). This study was granted approval by GPRD’s Independent Scientific Advisory Committee (ISAC).

## Results

### Patient characteristics

Out of 74,763 patients in the sample, 5870 (7.85%) patients who were registered at 445/457 general practices had a first-ever recorded diagnosis of cancer between 1999 and 2008. Of these patients, 13.9% were diagnosed during an emergency admission (n = 817/5870). Through this route and based on NCIN’s cancer categories, the three most common types of cancers were “other” (21.4%; n = 193/902), breast (13.6%; n = 123/902) and colorectal (11.5%; n = 104/902) cancers (Table 1) [22]. Patients diagnosed by emergency admission had up to two cancer diagnoses for that episode of care, with 1.45% of patients recorded as having dual first-ever cancer diagnoses (n = 85/5870). Cancers of the bone, connective tissue, skin and breast were most frequently recorded out of all cancer diagnosis codes recorded in both emergency and non-emergency routes (57.2%, n = 309/540). Due to imperfect matching between Read and ICD-10 codes, lower level categorisation was not possible.

**Table 1:** Number of diagnoses by cancer type for first cancer diagnoses by emergency admission, n = 902 diagnoses

**Cancer type**	**Frequency, n**	**(%)**
Other cancer	193	(21.4)
Breast	123	(13.6)
Colorectal	104	(11.5)
Lung	95	(10.5)
Prostate	84	(9.31)
Bladder	54	(5.99)
Non-hodgkin’s lymphoma	37	(4.10)
Oesophagus	31	(3.44)
Uterus	22	(2.44)
Kidney	21	(2.33)
Ovary	18	(2.00)
Stomach	17	(1.88)
Brain & central nervous system	16	(1.77)
Multiple myeloma	16	(1.77)
Pancreas	16	(1.77)
Oral	11	(1.22)
Melanoma	10	(1.11)
Chronic leukaemia	9	(1.00)
Acute leukaemia	8	(0.89)
Testis	8	(0.89)
Cervix	7	(0.78)
Larynx	2	(0.22)

### Incidence of cancer diagnosis by emergency admission

**Figure 1:**
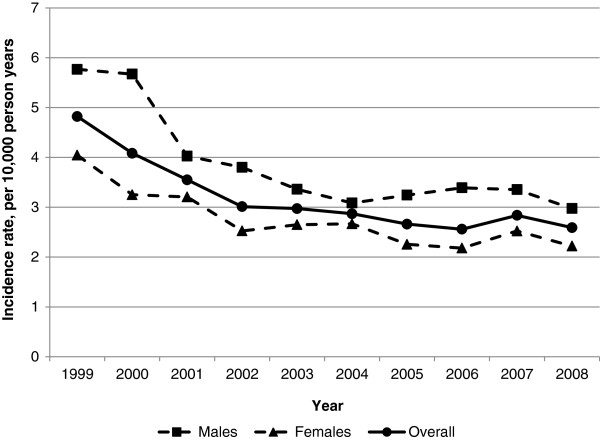
Incidence of first cancer diagnoses by emergency admission in England, by sex and year (1999-2008).

The incidence of first-ever recorded diagnoses of cancer by emergency admission during the study period was 2.51 patients per 10,000 person years. Over the ten years, the rate of cancer diagnoses by the emergency route declined, with a slight fluctuation in the penultimate year, 2007 (Figure 1). The incidence over the study period was higher in male patients but the greatest decrease in new cases was also in male patients, from 5.77 patients per 10,000 person years in 1999 to 2.98 patients per 10,000 person years in 2008. Compared with patients aged between 15 and 64 years old, the most marked decline in new cancer diagnoses by emergency admission was in patients aged 65 years or older, from 7.10 patients per 10,000 person years in 1999 to 3.94 patients per 10,000 person years in 2008. Yet, as Table 2 shows, almost half of first diagnoses by emergency admission were in patients aged between 65 and 84 years (48.2%, n = 494/817).

**Table 2:** Risk factors for first diagnosis of cancer, crude and adjusted results from Poisson regression using the generalized estimating equations (GEE) method

**Characteristic**	**All routes (n)**	**Emergency admission (n)**	**(%)**	**Crude**		**Adjusted**	
				**RR (95% CI)**	**P-value**	**RR (95% CI)**	**P-value**
**Age group at diagnosis (years)**					<.0001		<.0001
0–14	439	8	(0.98)	0.04 (0.02–0.08)	<.0001	0.11 (0.06–0.21)	<.0001
15–44	2347	61	(7.47)	0.06 (0.04–0.08)	<.0001	0.13 (0.10–0.17)	<.0001
45–64	1680	259	(31.7)	0.34 (0.27–0.42)	<.0001	0.52 (0.44–0.63)	<.0001
65–84	1197	394	(48.2)	0.72 (0.57–0.90)	0.004	0.73 (0.60–0.87)	0.001
≥85	207	95	(11.6)	1		1	
**Sex**					<.0001		-
Male	2418	400	(49.0)	1			
Female	3452	417	(51.0)	0.73 (0.64–0.84)	<.0001		
**Ethnicity**					<.0001		<.0001
Asian	29	6	(0.73)	4.48 (1.98–10.1)	<.0001	3.02 (1.67–5.48)	<.0001
Black	30	8	(0.98)	5.77 (2.84–11.7)	<.0001	2.09 (1.53–2.87)	<.0001
White	1924	617	(75.5)	6.94 (5.87–8.20)	<.0001	3.14 (2.48–3.98)	<.0001
Other	36	8	(0.98)	4.81 (2.37–9.76)	<.0001	3.06 (2.10–4.47)	<.0001
Unknown	3851	178	(21.8)	1		1	
**Deprivation**					<.0001		<.0001
Least deprived	716	132	(16.2)	2.95 (2.36–3.68)	<.0001	1.60 (1.27–2.02)	<.0001
Quintiles 2,3,4	1738	382	(46.8)	3.51 (2.95–4.19)	<.0001	1.81 (1.47–2.23)	<.0001
Most deprived	426	116	(14.2)	4.35 (3.45–5.49)	<.0001	1.93 (1.51–2.47)	<.0001
Unknown	2990	187	(22.9)	1		1	
**Charlson Index score**					<.0001		0.019
0	5785	745	(91.2)	1		1	
1	43	39	(4.77)	7.04 (5.10–9.72)	<.0001	1.34 (1.06–1.69)	0.014
≥2	42	33	(4.04)	6.10 (4.31–8.65)	<.0001	0.87 (0.70–1.07)	0.180
**Practice region**					<.0001		-
East Midlands	122	5	(0.61)	0.32 (0.13–0.79)	0.013		
East of England	835	140	(17.1)	1.31 (1.01–1.70)	0.041		
London	345	51	(6.24)	1.16 (0.82–1.63)	0.400		
North East	252	38	(4.65)	1.18 (0.81–1.72)	0.388		
North West	615	105	(12.9)	1.34 (1.01–1.77)	0.041		
South Central	763	92	(11.3)	0.94 (0.71–1.26)	0.695		
South East Coast	622	118	(14.4)	1.49 (1.13–1.95)	0.004		
South West	834	84	(10.3)	0.79 (0.59–1.06)	0.114		
West Midlands	746	90	(11.0)	0.95 (0.71–1.26)	0.699		
Yorkshire & The Humber	736	94	(11.5)	1			
**Time at practice (years)**					<.0001		-
Low	1957	176	(21.5)	1			
Moderate	1957	279	(34.2)	1.59 (1.31–1.91)	<.0001		
High	1956	362	(44.3)	2.06 (1.72–2.46)	<.0001		
**Follow-up time (years)**					<.0001		-
<1	988	82	(10.0)	0.45 (0.35–0.57)	<.0001		
1–3	2032	271	(33.2)	0.72 (0.60–0.85)	<.0001		
4–6	1571	226	(27.7)	0.77 (0.64–0.93)	0.006		
7–10	1279	238	(29.1)	1			
**Continuity of care**					<.0001		<.0001
Low	164	134	(16.4)	1		1	
Moderate	162	131	(16.0)	0.99 (0.78–1.26)	0.933	1.04 (0.90–1.20)	0.625
High	166	143	(17.5)	1.05 (0.83–1.33)	0.660	1.00 (0.87–1.15)	0.976
Not valid^*^	5378	409	(50.1)	0.09 (0.08–0.11)	<.0001	0.28 (0.24–0.33)	<.0001
**Consultation ≤30 day before diagnosis†**					<.0001		-
0	5734	704	(86.2)	1			
1	136	113	(13.3)	6.77 (5.55–8.25)	<.0001		
**Referral ≤30 days before diagnosis‡**					0.022		-
**Admission before diagnosis, mean (SD)**	0.04 (0.20)	0.12 (0.33)		0.30 (0.21–0.43)	<.0001	0.31 (0.20–0.46)	<.0001

^*^Patient had fewer than 2 consultations with a GP or nurse at the practice or by telephone during the study period.

†At practice, by telephone, or home visit, with a GP or nurse.

‡Results for dichotomous “referral ≤30 days before diagnosis” variable omitted due to small numbers.

### Crude risk factors for diagnosis by emergency admission

In unadjusted regression analyses, all included variables were predictive of diagnosis by emergency admission. Age at diagnosis, social deprivation status (where deprivation was known), length of time registered at the practice and follow-up time had positive relationships with diagnosis by the emergency route (Table 2). Female patients were less at risk of diagnosis by this route than male patients (RR 0.73, 95% CI 0.64 to 0.84; p < 0.0001). Compared with patients registered at practices in Yorkshire & The Humber, patients of practices in the East of England (RR 1.31, 95% CI 1.01 to 1.70; p = 0.041), North West (RR 1.34, 95% CI 1.01 to 1.77; p = 0.041) or the South East coast (RR 1.49, 95% CI 1.13 to 1.95; p < 0.004) were most at risk of a first-ever cancer diagnosis by emergency admission. Patients with a moderate comorbidity score as measured by a total Charlson Index score of 1 were at higher risk compared with patients who had a score of 0 (RR 7.04, CI 5.10 to 9.72; p < 0.0001).

### Adjusted risk factors for diagnosis by emergency admission

As comorbidity is an important factor in cancer diagnosis and treatment, the stepwise model that included this variable was considered the final adjusted model, containing 6 out of the original 12 risk factors of interest. Once adjusted for other factors and taken into account clustering of patients at general practices, patient’s age at diagnosis, ethnicity, deprivation status, comorbidities (Charlson Index score), continuity of care and number of all-cause emergency admissions before diagnosis remained significant predictors of first-ever cancer diagnosis by the emergency route.

Two of the 6 patient characteristics continued to display the monotonic trends detected in crude analyses: the risk of diagnosis by emergency admission increased with age and deprivation, p < 0.0001 for each variable. Compared to patients with unknown ethnicity status, patients of white ethnicity remained at greatest risk of diagnosis by emergency admission (RR 3.14, 95% CI 2.48 to 3.98; p < 0.0001). Patients with a moderate comorbidity score, measured by a total Charlson score of 1 compared to a score of 0, also remained more at risk of cancer diagnosis by the emergency route (RR 1.34, 95% CI 1.06 to 1.69; p = 0.014).

### Service use

Within the 30 days immediately before diagnosis, 0.46% of patients diagnosed via non-emergency routes had one or more consultations with a GP or nurse at their general practice, by telephone or home visit (mean 0.01, SD 0.08 consultations) compared with 13.8% of patients diagnosed by emergency admission (mean 0.25, SD 0.81 consultations), p < 0.0001. Although this factor was crudely associated with the risk of diagnosis by emergency admission, it was no longer statistically significant once adjusted for other factors and clustering of patients at practices (p = 0.124). Regardless of the route to diagnosis, the numbers of all-cause emergency admissions and referrals before the first cancer diagnosis and during the study period were low, with fewer than 5 patients recorded as having one or more referrals in the 30 days prior to diagnosis. In patients diagnosed via the emergency route, 3.3% had at least one prior admission during the study period (n = 27/817, maximum 2 admissions per patient) compared with 12.3% of patients diagnosed via non-emergency routes (n = 619/5053, maximum 3 admissions per patient), p < 0.0001. After adjustment, the number of prior emergency admissions remained inversely associated with risk of subsequent diagnosis through the emergency route (RR 0.31 per prior emergency admission, 95% CI 0.20 to 0.46; p < 0.0001).

## Discussion

### Summary of findings

In this cross sectional observational study, we explored temporal trends in first-ever cancer diagnoses by emergency admission in England between 1999 and 2008. Adjusted analyses highlighted patient groups at greater risk of diagnosis by this route. These groups included older patients and those living in the most deprived areas, as found in other studies [9,29]. Even though the number of patients who had at least one previous all-cause emergency admission was small, this characteristic was associated with lower risk of subsequent cancer diagnosis by the emergency route. Unlike previous studies, we did not find regional variation in diagnoses by emergency admission once adjusted for other factors [11]. Encouragingly, we found a reduction in the rate of first cancer diagnoses by the emergency route over time, which suggests that initiatives to improve cancer awareness in primary care and to improve access to cancer services may be having positive effects.

### Comparison with other studies

Our finding that 13.9% of diagnoses were made during an emergency admission is similar to the estimate of 12.9% from the National Audit of Cancer Diagnosis in Primary Care which also included emergency referrals, and 16.4% of oesophagogastric cancers by Palser et al., 2013 [9,29,30]. However, our finding is lower than the estimate of 24% from NCIN, which was calculated from a combination of HES, cancer registry, and screening data as well as Cancer Waiting Times, and our earlier study which used HES data [9-11]. The discrepancy between earlier estimates and ours here may be due to differences in case assignment (diagnosis during two or three years compared with first-ever diagnosis, respectively) [31]. Compared to other sources such as cancer registries, GPRD data have produced a lower incidence of cancer but the GPRD also appears to contain valid and reliable records of cancer diagnoses [19,20].

Previous studies have found regional variations in the incidence of cancer and patient outcomes but in this study we found no statistically significant difference by practice region in the risk of diagnosis by emergency admission [4,9,12]. Our finding may be due to the relatively small sample size and more research is required for validation. Similarly, sex was also no longer statistically significant in adjusted regression analyses. This finding may again be due to the sample size or the cancer types included in the study. With known sex-differences in risk and outcomes by different types of cancers, further research and especially studies by cancer type should continue to consider sex as a potential predictor [8,11,16,20].

Poor access to primary care services is one explanation for delayed diagnoses including those made by the emergency route, although the relationship is likely to be complex [4,11,32]. While the number of consultations with GPs or nurses in the 30 days immediately before diagnosis was not a statistically significant risk factor once all other factors had been considered, a much greater proportion of patients diagnosed by emergency admission also consulted with a GP or nurse in the preceding 30 days compared with patients diagnosed by non-emergency routes (13.8% compared with 0.46%, respectively). Lyratzopoulos et al. (2012) also found variation in pre-referral consultations by age, sex, ethnicity, and cancer type [8]. Further exploration of this phenomenon in patients with potentially delayed or untimely and/or late diagnosis is warranted to determine if “alarm” symptoms are being overlooked [16].

### Strengths and limitations

Our study benefitted from the use of linked primary and secondary care data combined with mortality data from the GPRD. Cancer registry data are typically considered the gold standard for research on cancer incidence and patient outcomes. However, both GPRD and HES data have shown comparable completeness to cancer registries [19,33]. The integrated data are likely to improve the accuracy of patient identification beyond that achieved in previous studies [11,13,14,29,34]. We applied rigorous sampling criteria, tracking back to patients’ first records and cross-referenced between data from primary and secondary care, to capture patients’ first-ever diagnoses. Our sampling method is therefore more specific than other studies that used shorter timeframes for case ascertainment, such as Raine et al. (2010) and Bottle et al. (2012) who used one-year and three-year look-back periods respectively [11,13]. Furthermore, the United Kingdom offers universal health coverage to its population, including registration with a GP, and people can only be registered with one general practice at a time [35]. This makes the data collected by GPs in their electronic patient records a very valuable resource for research [36].

Limitations of this study include the lack of analysis by cancer type, which would be useful to inform clinical practice and management, especially given known differences in symptoms, treatments and prognoses between cancers. We performed our analyses at the patient level and not by cancer type because a minority of patients had dual first-ever diagnoses. Another reason for this approach was the lack of precise cross-mapping between Read codes and ICD-10 codes for cancer diagnoses that are required for analyses by cancer type. A further methodological consideration is that despite facilitating the accurate capture of first-ever diagnoses, our sampling method prevented the inclusion of patients diagnosed with secondary or recurrent cancers and patients not diagnosed with cancer for the first-ever time. However, the service-seeking behaviours and care pathways of patients defined as such may differ from patients with a first-ever diagnosis of cancer. Thus, it was appropriate for this study to exclude these patients, but these patient groups may be of interest for further study. Our findings may also be affected by missing data for some variables, such as ethnicity and social deprivation, although the risk factors identified in this study are supported by empirical evidence. As the modified Charlson Index was mapped using an ICD-9-CM based scoring method, there may have been inaccurate and incomplete comorbidity matching to Read and ICD-10 diagnosis codes in this study. This may be one explanation for the relatively low number of patients with recorded comorbidity.

We applied stepwise selection despite known limitations of this procedure, such as sensitivity to variable ordering and combinations. To select the final regression model, we considered a full model and those using forward, backward and interactive-forward approaches. There was little difference between the models in fit or the variables retained, in support of the appropriate use of stepwise selection in this study. The interval between initial primary care presentation and referral for the four main cancers (breast, colorectal, lung and prostate) is longer than 30 days for a large proportion of diagnosed patients [29,37]. For this reason, a longer time interval, such as a window of the prior 3 months, may be more suitable when examining patterns in consultations preceding cancer diagnosis [1,37]. Referral rates for suspected cancer differ across the country [12,29,38,39]. Unfortunately, the quality of related data was poor in the study dataset and little interpretation of the results for referrals can be made without validation using other data sources.

## Conclusions

Despite robustness in case identification from the linked data, to better understand why some patient groups are more susceptible to delayed and potentially late diagnosis by emergency admissions, cancer staging and treatment data are needed [38]. Further data on cancer screening and waiting times between intervals of care will help to establish which groups are most affected by untimely referral and/or diagnosis, and also identify which elements of the care pathway should be targeted for improvement [10].

Our population-based analyses have used recent data to determine a range of patient characteristics associated with first-ever diagnoses of cancer by the emergency route as a proxy measure of delayed diagnosis. The results contribute to national efforts to improve cancer care in England by increasing understanding of patient groups at high risk of untimely diagnosis [9,29,40]. This research focused only on the beginning of the patient cancer pathway and attention must also be given to the entire continuum, including better methods of identifying alarm symptoms in primary care, referral for specialist care, and long-term patient outcomes.

## Abbreviations

A&E: Accident and emergency; COC: Continuity of care index; CPRD: Clinical practice research datalink; GEE: Generalized estimating equation method; GP: General practitioner; GPRD: General practice research database; HES: Hospital episode statistics; IMD: Index of multiple deprivation; ISAC: Independent scientific advisory committee; MRC: Medical research council; NCIN: National cancer intelligence Network; NHS: National health service; ONS: Office for national statistics; RR: Relative risk.

## Competing interests

The authors declare that they have no competing interests.

## Authors’ contributions

CT, AB, and PA developed the study. CT carried out the statistical analyses. All authors contributed to the interpretation of the data, CT drafted the paper. All authors revised the manuscript. All authors read and approved the final manuscript.

## Pre-publication history

The pre-publication history for this paper can be accessed here:

http://www.biomedcentral.com/1472-6963/13/308/prepub

## Supplementary Material

Additional file 1: Table S1 Excluded cancer diagnoses, ICD-10 codes mapped to Read codes. Click here for file

Additional file 2: Table S2 Cancer diagnoses, ICD-10 codes mapped to Read codes. Click here for file
