# Supplementary material for: Cancer diagnosed by emergency admission in England: an observational study using the general practice research database
Source: BMC Health Serv Res. 2013 Aug 14;13:308. doi: 10.1186/1472-6963-13-308 (PMC3751722; doi:10.1186/1472-6963-13-308)
Supplement: Additional file 1: Table S1 — Excluded cancer diagnoses, ICD-10 codes mapped to Read codes. [file 1472-6963-13-308-S1.docx]

**Additional file 1**

Supplementary Table S1.

Excluded cancer diagnoses, ICD-10 codes mapped to Read codes

| **ICD-10 code** | **Read code** | **Cancer type** |
| --- | --- | --- |
| C44 | B33.. | Other malignant neoplasms of skin |
| C97 | ByuE | Malignant neoplasms of independent (primary) multiple sites |
| *D00-D09* | *B8…, ByuF.* | *In situ neoplasms* |
| *D10-D36* | *B7…, ByuG.* | *Benign neoplasms* |
| *D37-D48* | *B9…, BA…, ByuH.* | *Neoplasms of uncertain or unknown behaviour* |

Source: Health and Social Care Information Centre: **UK Terminology Centre - Read Codes**. http://systems.hscic.gov.uk/data/uktc/readcodes/index_html

National Cancer Intelligence Network: **Routes to Diagnosis, 2006-2008. NCIN technical document**. London, National Cancer Intelligence Network; 2010.

NHS Health and Social Care Information Centre: **NHS Clinical Terminology Browser Version 1.04**.
