# Supplementary material for: Cancer diagnosed by emergency admission in England: an observational study using the general practice research database
Source: BMC Health Serv Res. 2013 Aug 14;13:308. doi: 10.1186/1472-6963-13-308 (PMC3751722; doi:10.1186/1472-6963-13-308)
Supplement: Additional file 2: Table S2 — Cancer diagnoses, ICD-10 codes mapped to Read codes. [file 1472-6963-13-308-S2.docx]

**Additional file 2**

Supplementary Table S2.

Cancer diagnoses, ICD-10 codes mapped to Read codes

| **ICD-10 code** | **ICD-10 term** | **Read code** | **Read term** |
| --- | --- | --- | --- |
| C00-C14 | Malignant neoplasms of lip, oral cavity and pharynx | B0… | Malignant neoplasm of lip, oral cavity and pharynx |
| C15-C26 | Malignant neoplasms of digestive organs | B1… | Malignant neoplasm of digestive organs and peritoneum |
| C30-C39 | Malignant neoplasms of respiratory and intrathoracic organs | B2... | Malignant neoplasm of respiratory tract and intrathoracic organs |
| C40-C41 | Malignant neoplasms of bone and articular cartilage | B3... | Malignant neoplasm of bone, connective tissue, skin and breast |
| C43-C44 | Melanoma and other malignant neoplasms of skin | B3... | Malignant neoplasm of bone, connective tissue, skin and breast |
| C45-C49 | Malignant neoplasms of mesothelial and soft tissue | B3... | Malignant neoplasm of bone, connective tissue, skin and breast |
| C50-C50 | Malignant neoplasm of breast | B3... | Malignant neoplasm of bone, connective tissue, skin and breast |
| C51-C58 | Malignant neoplasms of female genital organs | B4... | Malignant neoplasm of genitourinary organ |
| C60-C63 | Malignant neoplasms of male genital organs | B4... | Malignant neoplasm of genitourinary organ |
| C64-C68 | Malignant neoplasms of urinary tract | B4... | Malignant neoplasm of genitourinary organ |
| C69-C72 | Malignant neoplasms of eye, brain and other parts of central nervous system | B5... | Malignant neoplasm of other and unspecified sites |
| C73-C75 | Malignant neoplasms of thyroid and other endocrine glands | B6... | Malignant neoplasm of lymphatic and haemopoietic tissue |
| C76-C80 | Malignant neoplasms of ill-defined, secondary and unspecified sites | B5... | Malignant neoplasm of other and unspecified sites |
| C81-C96 | Malignant neoplasms, stated or presumed to be primary, of lymphoid, | B6... | Malignant neoplasm of lymphatic and haemopoietic tissue |

Source: Health and Social Care Information Centre: **UK Terminology Centre - Read Codes**. http://systems.hscic.gov.uk/data/uktc/readcodes/index_html

National Cancer Intelligence Network: **Routes to Diagnosis, 2006-2008. NCIN technical document**. London, National Cancer Intelligence Network; 2010.

NHS Health and Social Care Information Centre: **NHS Clinical Terminology Browser Version 1.04**.
